# Collagen cross-linking impact on keratoconus extracellular matrix

**DOI:** 10.1371/journal.pone.0200704

**Published:** 2018-07-18

**Authors:** Rabab Sharif, Ben Fowler, Dimitrios Karamichos

**Affiliations:** 1 Department of Cell Biology, University of Oklahoma Health Sciences Center, Oklahoma City, OK, United States of America; 2 Oklahoma Medical Research Foundation (OMRF), Imaging Core Facility, Oklahoma City, OK, United States of America; 3 Department of Ophthalmology/Dean McGee Eye Institute, University of Oklahoma Health Sciences Center, Oklahoma City, OK, United States of America; University of Missouri-Columbia, UNITED STATES

## Abstract

**Background:**

Keratoconus (KC) is a common multifactorial ectatic corneal disease with unknown onset. KC most commonly appears in adolescence and affects approximately 1:400 people worldwide. Treatment options, for advanced KC cases, are collagen cross-linking (CXL) and corneal transplants. CXL is a new KC treatment that helps arrest the disease. Unfortunately, only a fraction of KC patients will qualify for CXL treatment. Our goal, in this study, was to begin to understand how CXL affects the corneal microenvironment and pave the way towards a more patient-driven CXL treatment.

**Methods:**

Primary human corneal fibroblasts from healthy and KC donors were plated on transwell polycarbonate membranes and stimulated by a stable vitamin C. At 4 weeks, riboflavin was added followed by UVA irradiation. Transmission Electron Microscopy (TEM) and western blots were used to assess the effect of CXL on the extracellular matrix (ECM) and the resident cells, pre- and post CXL.

**Results:**

Data shows CXL improved lamellar organization showing more organized collagen fibrils decorated with proteoglycans (PGs). The distribution of the collagen fibrils and interfibrillar spacing was also visibly improved, post-CXL. Lumican, mimecan, and decorin were the dominant PGs and were significantly upregulated in post-CXL cultures. ECM degradation proteins, matrix metalloproteinases (MMPs), MMP-1, -3, and -9, but not MMP-2, were significantly downregulated post-CXL. TIMP-1 and -2 were not modulated by CXL.

**Conclusion:**

The unknown effects of CXL on the human corneal microenvironment have hampered our ability to make CXL available to all KC patients. Our current study provides a deeper understanding on CXL activity, using our unique 3D *in vitro* model.

## Introduction

Keratoconus (KC) is a common ectatic corneal disease which impairs vision by causing corneal thinning, bulging and scarring [1]. Clinical findings include discomfort, visual disturbance, and possible blindness if left untreated [2]. KC is known to affect approximately 1:400 to 1:2000 people worldwide [3]. Initially, spectacles and rigid gas permeable lenses are used to correct the vision of KC patients [4]. As the disease progresses and refraction correction is no longer possible, the treatment for KC has traditionally been penetrating keratoplasty (PK) [5].

In recent years, collagen cross-linking (CXL) has emerged as a minimally invasive treatment option to arrest the progression of KC [6, 7]. According to *Global Consensus of Keratoconus and Ectatic Diseases*, 83.3% of ophthalmic physicians are performing CXL as a KC treatment [8]. To-date a large number of studies have evaluated the short and long-term effectiveness of CXL, revealing contradictory results.

Although CXL is commonly used in the clinics, a number of alternative techniques have been proposed in order to overcome current complications, such as large epithelial defects that occur because of the epithelial removal prior to CXL treatment. These defects vary, vastly, from minor to more devastating, including infectious keratitis and corneal perforations [9, 10]. Another CXL drawback is the prolonged treatment time that is required based on the Dresden protocol. Studies on CXL effectiveness have illustrated variable and sometimes adverse outcomes, such as worsening of topographic and pachymetric values [11, 12]. Perhaps the most important drawback related to CXL is the fact that it is not suitable for every single patient with KC. Currently, the best candidate to receive therapy are patients with progressive KC, but who also satisfy the following criteria: patients between the ages 16 and 40 years, with a minimum corneal thickness of 400 microns, maximal keratometry <60 D (Pentacam readings), and have no other known corneal disease. CXL is, therefore, not the ultimate treatment for KC, since it is not suitable for every KC patient.

It is well documented that the vast majority of the published studies are focusing on the biomechanical effects of CXL, neglecting critical information such as ultrastructural and microenvironment alterations, cellular responses, and reactive oxygen species generation. Recently, our group introduced a 3D cell-based CXL model in order to determine and quantify the effect of CXL at the cellular and molecular level [13]. This groundbreaking work, paves the way for a deeper understanding on how CXL works and future refinement in the CXL technique to allow for more targeted clinical procedures. In the current study, we further characterize our novel 3D CXL *in vitro* model by reporting the microenvironment alterations, caused by CXL.

## Methods

### Ethics

All procedures used in this study adhered to the tenets of the Declaration of Helsinki. Healthy human corneas were obtained from the National Disease Research Interchange (NDRI, Philadelphia, PA). KC donor corneas were obtained from our clinical collaborators Drs. Hjortdal (Aarhus University Hospital, Aarhus, Denmark), and Garett (Dean McGee Eye Institute, Oklahoma City, OK). Patient written informed consent was obtained and research protocols were approved prior to initiation of experiments reported in this study. Institutional review board (IRB) approval was obtained at the University of Oklahoma Health Sciences Center—Dean McGee Eye Institute,IRB protocol #3450 and research protocol # 1-10-72-77-14 was approved by the central Denmark region committee on health research ethics Inclusion criteria for healthy controls required, absence of KC diagnosis or other corneal diseases. Inclusion criteria for KC patients required diagnosis of KC by a certified ophthalmologist and absence of other ophthalmic conditions, and to exclude patients who had previously received CXL or undergone PK.

### Corneal tissue processing and cell isolation

Primary corneal fibroblasts from Healthy and KC human corneas were isolated and processed as previously described [14]. Through brief scraping with a razor blade, the endothelium and epithelium were removed from the stroma. The stromal tissue was cut into small pieces (4 to 5 pieces of 2 mm × 2 mm). Stromal tissue pieces were allowed to adhere to the bottom of a T25 flask for 30 minutes at 37 °C before carefully adding Eagle’s Minimum Essential Media (EMEM: ATCC: Manassas, VA) containing 10% Fetal Bovine Serum (FBS: Atlantic Biologic’s; Lawrenceville, CA) and 1% Antibiotic/Antimycotic solution (Gibco® Antibiotic-Antimycotic, Life technologies, Grand Island, NY) to the flask without disturbing the explants. At 80–90% confluency, explants were further passaged into T75 flask and incubated at 37 °C, 5% CO_2_ for further expansion and analysis.

Donor information for Human Corneal Fibroblasts (HCFs) utilized in this study: N22 (69y/o male), N24 (43y/o female), and N4 (53 y/o, male). Donor information for Human Keratoconus Cells (HKCs) utilized in this study: GF24 (60 y/o male), WU2 (69 y/o male), and GF28 (38 y/o female).

### 3D model and ECM assembly

The 3D *in vitro* model has been previously described extensively [13–15]. Briefly, HCFs and HKCs were seeded on transwell 6-well plates with polycarbonate membrane inserts with 0.4-μm pores (Transwell; Corning Costar; Charlotte, NC) at a density of 1 × 10^6^ cells/well and cultured in 1.5 mL of 10% FBS EMEM medium and 1% Antibiotic, stimulated with 0.5 mM 2-O-α-D-Glucopyranosyl-L-Ascorbic Acid (Vitamin C, American Custom Chemicals Corporation, San Diego, CA) in both top and bottom wells. Fresh media was supplied every other day for the duration of 4 weeks to promote native ECM assembly [14].

### 3D CXL model

The 3D CXL model was recently developed [13], establishing a setup that mimicks the current clinical CXL protocol. Briefly, both cell types (HCFs and HKCs) were plated on our 3D transwell polycarbonate membrane inserts, as previously described [14]. At week 4, a mixed riboflavin 0.1% PBS solution was added to the constructs followed by UVA irradiation. Using a UV-X illumination system (version 1000; IROC AG, Zurich, Switzerland) [13], at a wavelength of 360–370 nm and an irradiance of 3 mW/cm2 of UVA, with a total energy dose of 5.4 J/cm^2^ [16, 17]. This UV-X illumination system was calibrated prior to each treatment using a UVA meter (LaserMate-Q; LASER 2000, Wessling, Germany). Each well was exposed to UVA for 3 minutes at a 3 cm distance, mirroring CXL clinical settings [16, 18]. Post irradiation, each construct was rinsed 3X with PBS and incubated in fresh media for 12 h to promote post radiation repair, before any further analysis was performed.

### TEM

After 4 weeks in culture, 3D cell cultures were fixed while covered and on a rocker with 4% Paraformaldehyde (EM grade), 2% Gluteraldehyde (EM grade), in 0.1M Sodium Cacodylate buffer for 1 day at 4⁰C. Samples were then post fixed for 90 minutes in 1% Osmium tetroxide (OsO4) in Sodium Cacodylate, and rinsed three times for five minutes each in 0.1M Sodium Cacodylate buffer. The samples were then dehydrated in a graded acetone series. Then the cells had two 15 minute treatments in 100% Propylene Oxide. Following dehydration, the samples were infiltrated in a graded Epon/Araldite (EMS) resin /Propylene Oxide series (1:3, 1:1, 3:1) for 60 minutes,120 minutes, and overnight respectfully. The following day samples were further infiltrated with pure resin for 45 minutes, 90 minutes, and then overnight. The cell constructs were then embedded in resin plus BDMA (accelerator) and polymerized at 60°C for 48 hours. Ultrathin sections were stained with Lead Citrate and Uranyl Acetate before viewing on a Hitachi H7600 Transmission Electron Microscope at 80 kV equipped with a 2k X 2k AMT digital camera.

### Western blots

Western blot (WB) analyses of both HCFs and HKCs were performed, as previously described [13, 19]. Protein concentration was assessed using Bradford assay (Thermo Scientific, IL). 4–20% Tris-Glycine gels (Novex, Life technologies, Carlsbad, CA) was used for gel electrophoresis, to which equal amounts of proteins were loaded and a protein transfer was done using Nitrocellulose membrane (Novex, Nitrocellulose membrane filter par sandwich, Life Technologies). After incubation in a 5% BSA blocking solution (Thermo Scientific, IL), the membranes were incubated with 1:500–1:1000 dilutions of primary anti-human rabbit antibodies Keratocan (ab113115; Abcam, Cambridge, MA, USA), Mimecan (ab110558; Abcam, Cambridge, MA), Decorin (ab175404; Abcam, Cambridge, MA), Lumican (ab98067; Abcam, Cambridge, MA), MMP1 (ab38929; Abcam, Cambridge, MA), MMP2 (ab37150; Abcam, Cambridge, MA), MMP3 (ab53015; Abcam, Cambridge, MA), MMP9 (ab38898; Abcam, Cambridge, MA), TIMP1 (ab61224; Abcam, Cambridge, MA) and TIMP2 (ab53730; Abcam, Cambridge, MA). After primary incubation, the membrane was washed for 5 min (3×) in Tris-buffered Solution with Tween20 before probing with secondary antibody Goat anti-Rb Alexafluor 568 (Life Technologies, Grand Island, NY, USA) at 1:2000 dilution for 1 h with rocking at room temperature. The membrane was allowed to dry before imaging using ChemiDoc-it to image. GAPDH (ab9485; Abcam, Cambridge, MA) was used as the loading control and results were analyzed by normalizing the value to that of the loading control expression and plotting the fold expression.

### Statistical analysis

GraphPad Prism 7.02 software was used for statistical analysis and a one-way ANOVA was performed. P≤ 0.05 was considered statistically significant. The n number for each experiment is listed in the appropriate figure legend. All graphs show mean ± standard error of the mean.

## Results

### Ultrastructure alterations

*In vivo*, CXL is known to improve corneal curvature along with topographic and visual parameters. In this study, we examined the ECM alterations following CXL treatment *in vitro*.

**Fig 1:**
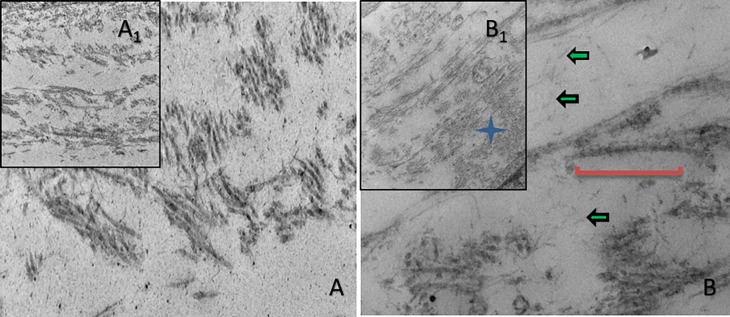
TEM image showing corneal matrix status. HCF.C and HKC.C: pre-CXL, and HCF.X and HKC.X: post-CXL, when cultured in our 3D model. Green arrows: proteoglycans; Blue star: collagen fibrils organization; Red line: collagen banding.

Our data shows that the CXL treatment improved the overall organization of the lamellar structure of the CXL cultures (Fig 1B and 1B_1_). Collagen fibrils emerged more organized and running parallel to each other (Fig 1B_1_; blue star) compared to the pre-CXL cultures that showed a more random and sparse orientation (Fig 1A and 1A_1_). The collagen fibrils of the CXL cultures were also decorated with proteoglycans (Fig 1B; green arrows), something that was largely absent in the pre-CXL cultures. The distribution of the collagen fibrils, interfibrillar spacing, and collagen bands were also visibly improved by the CXL treatment (Fig 1B; red line).

### Proteoglycans

The corneal stroma constitutes 90% of the corneal thickness and is primarily composed of collagen fibrils that are coated by different PGs. These PGs are a diverse group of glycoconjugates composed of various core proteins post-translationally modified with anionic polysaccharides called glycosaminoglycans (GAGs). There are five major PGs located in the corneal ECM; lumican, keratocan, osteoglycin, decorin, and biglycan. Despite the importance of PGs with regards to the maintenance of the corneal integrity, there are very few studies examining alterations of PGs in connection with KC [2, 20, 21]. There are no studies on PGs and their modulation following CXL in KC.

**Fig 2:**
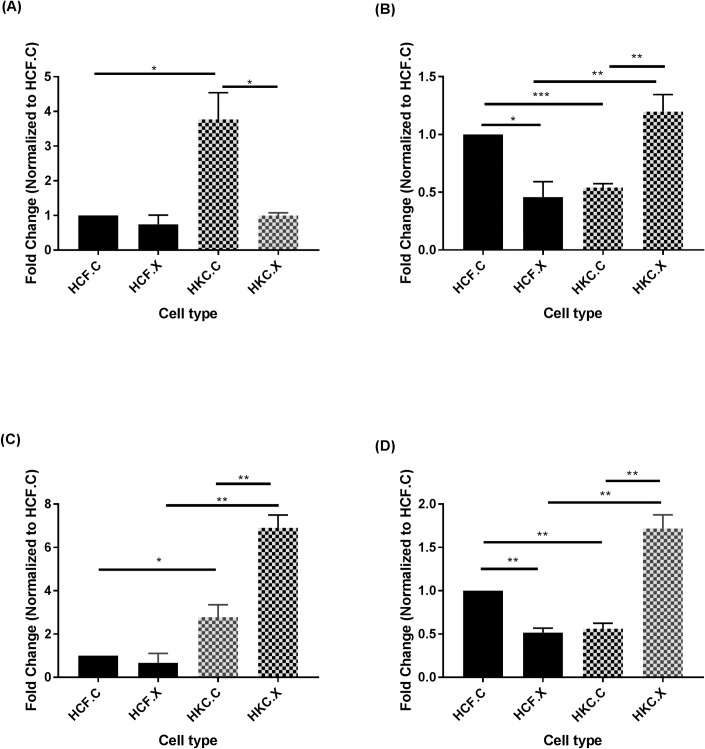
**CXL alters protein expression of PGs**: (A) Keratocan, (B) Lumican, (C) Mimecan, and (D) Decorin, in HCF.C and HKC.C: pre-CXL, and HCF.X and HKC.X: post-CXL. n = 4. Statistical significance was determined by one way ANOVA, with p ≤ 0.05 considered statistically significant. Error bars represent standard error of the mean *p<0.05, **p<0.01, ***p<0.001.

In this study, we investigated the protein expression of keratocan, mimecan, decorin, and lumican (Fig 2). 3D HKCs pre-CXL expressed high levels of keratocan that was downregulated to HCF levels following CXL (Fig 2A). On the other hand, Lumican, Mimecan, and Decorin were significantly upregulated following CXL, compared to pre-CXL (Fig 2B, 2C, and 2D respectively).

### Enzymatic degradation

The KC cornea is characterized by disturbed regulation of the corneal microenvironment that favors enzyme activity imbalance [22–24]. These enzymes include lysyl oxidases (LOX) and MMPs. In our recent study [13] we showed the significant upregulation of LOX following CXL, *in vitro*. Here, we investigated the modulation of several MMPs, since they are tightly linked to KC and the degradation of the corneal ECM.

**Fig 3:**
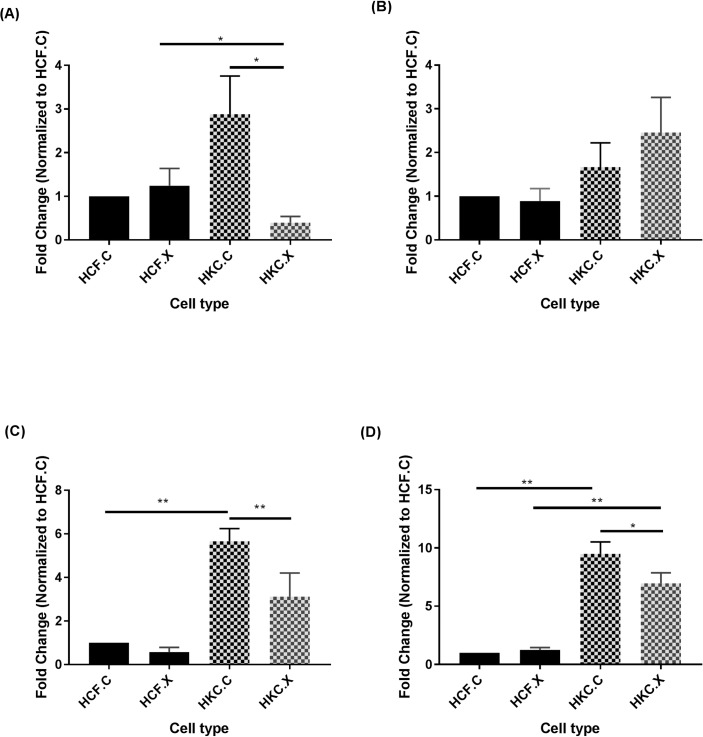
**CXL alters protein expression of MMPs**: (A) MMP1, (B) MMP2, (C) MMP3, and (D) MMP9, in HCF.C and HKC.C: pre-CXL, and HCF.X and HKC.X: post-CXL. n = 4. Statistical significance was determined by one way ANOVA, with p ≤ 0.05 considered statistically significant. Error bars represent standard error of the mean *p<0.05, **p<0.01.

Fig 3 shows protein expression of MMP-1, MMP-2, MMP-3, and MMP-9 in 3D HCF and HKC cultures pre and post-CXL. Data from pre-CXL HKCs showed significantly higher expression of MMP-3 and MMP-9 when compared to pre-CXL HCFs (Fig 3C and 3D respectively). Upon CXL, MMP-1 (Fig 3A), MMP-3 (Fig 3C), and MMP-9 (Fig 3D) were significantly downregulated in HKCs, indicating that CXL is positively affecting/ arresting the ECM degradation process. No differences were found in MMP-2 expression (Fig 3B). CXL did not alter the expression of any of the MMPs in HCFs suggesting healthy status and no ECM degradation onset. These findings could be correlated with some studies reporting regulation of MMPs in KC tear fluid [25–27]. However, this model remains the only *in vitro* model available for the studies of CXL-related on KC-derived cells/ECM.

**Fig 4:**
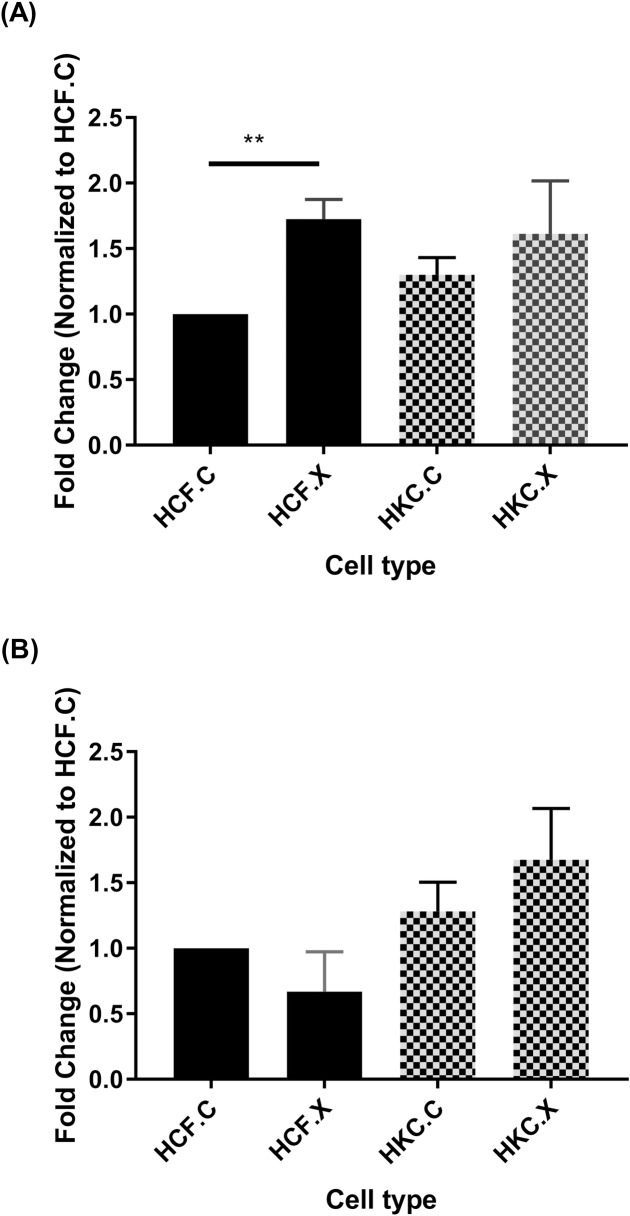
CXL shows no significant impact on protein expression of TIMPs in KC. (A) TIMP1, (B) TIMP2, in HCF.C and HKC.C: pre-CXL, and HCF.X and HKC.X: post-CXL. n = 4. Statistical significance was determined by one way ANOVA, with p ≤ 0.05 considered statistically significant. Error bars represent standard error of the mean **p<0.01.

Furthermore, we investigated the expression of tissue inhibitors of metalloproteinases (TIMPs). TIMPs are known for their inhibitory role against most of the known MMPs [28–30]. Our data shows that both TIMP-1 and TIMP-2 were not modulated in pre or post-CXL HKCs. However, TIMP-1 expression was significantly upregulated in HCFs following CXL (Fig 4A and 4B).

Collectively, our data suggests that MMPs are significantly upregulated in KC and their activity is inhibited by CXL, contributing to the arrest of KC progression *in vivo*. TIMP-1 and TIMP-2 may not be critical players in remodeling the KC corneal microenvironment.

## Discussion

CXL or else known as the Dresden protocol was introduced in 2003 [16] and is currently considered the standard KC treatment. CXL requires removal of the epithelial layer followed by 30 minutes riboflavin application and 30 minutes UVA irradiation [16, 31]. CXL also requires that the riboflavin solution is applied every 3–5 minutes for the duration of the irradiation. While CXL is accepted as the standard protocol for CXL, the need for corneal epithelial debridement (for maximum riboflavin penetration) is accompanied by pain and discomfort [32, 33]. Epithelial debridement also leads to total corneal thickness reduction, with potential extremely unwanted clinical problems in thin corneas [34, 35].

Long-term stabilization and vision improvement, following CXL, have been reported in many prospective studies [36, 37]. Unfortunately, failures and progression of keratectasia have also been reported, as a result of CXL. The best candidates for CXL are patients with progressive KC, but that is not the only criteria [38]. Candidates for CXL should also be between the ages 16 and 40 years, with a minimum corneal thickness of 400 microns, maximal keratometry <60 D (Pentacam), and have no other known corneal disease. Furthermore, very little information is known in terms of CXL long term safety and efficacy. It is easily understood that CXL is not the ultimate treatment for all KC patients. Ideally, CXL treatment parameters should be tailored towards patients and we should aim towards personalized treatment that will include all KC severities.

It is therefore essential to elucidate the precise molecular effects of CXL, not only on the exposed resident cells but also on the ECM components. Such an approach, will allow us be able to modify and improve the efficacy of CXL treatment. Considering there is currently no acceptable animal model for KC, our study and unique CXL *in vitro* model becomes even more relevant. Our data shown here provides significant insights on what is happening to corneal stromal cells as well as the ECM following CXL.

Previous KC studies, including ours, have documented that the disruption of this homeostasis is due to an upregulation of the degradative enzymes and a downregulation of their inhibitors [2, 39]. It is thought that this disruption may mediate the corneal thinning seen in KC [40]. MMPs have been reported to be highly modulated in KC [41]. Under normal physiological conditions, MMPs are responsible for tissue remodeling and degradation of the ECM [42, 43]. Many studies have reported altered expression of MMPs in KC. MMP-9 is shown to be significantly overexpressed in patients’ corneal epithelial cells as well as in tears [26, 44]. Elevated expressions of MMP-1, MMP-3, MMP-7, and MMP-13 in KC patients’ tears have also been shown [45, 46]. Our results show that MMP-3 and MMP-9, but not MMP-2, were elevated in HKCs compared to HCFs suggesting that MMP-3 and -9 may play a significant role in KC. Following CXL, all MMPs were downregulated, except for MMP-2, highlighting the effectiveness of shutting down enzymatic degradation. On the other hand, TIMP1 and TIMP2 were not significantly regulated by CXL, in HKCs. This agrees with what we know about the role of TIMPs in ECM homeostasis; where TIMPs are known to counteract MMPs activity [29, 30]. If MMPs are shutting down, following CXL, then there is no real need for TIMPs to respond or be modulated. Collectively, our data also suggests that deactivation of MMP expression via CXL treatment may contribute to ECM stability and homeostasis, which is critical in KC.

The transparency of the human cornea is known to be regulated by the uniform distribution of the collagen fibrils as maintained by PGs [47, 48]. Lumican, keratocan, and mimecan are known to carry a keratin sulfate chain [49] where decorin and biglycan carry a chondroitin sulfate chain [50]. The studies investigating modulation of PGs in KC are surprisingly limited [2, 51]. Studies reporting the effects of CXL in the human cornea and its effects on PGs are even sparser [52, 53]. Our study attempts to determine how CXL modulates PGs expression using our *in vitro* model. Our data shows significant upregulation of keratocan and mimecan and downregulation of decorin and lumican in HKCs when compared to HCFs, pre-CXL. Upon CXL, mimecan, decorin, and lumican were all significantly upregulated, in HKCs. Keratocan on the other hand was significantly downregulated, in HKCs. Previous studies have reported increased levels of keratocan in KC corneas, however little is known about its expression following CXL [54–56]. Zhang et al (2011) reported that keratocan and lumican interact with collagen in a different manner compared to mimecan and decorin [57]. While the authors used an *ex vivo* model, the observation is intriguing. The increased level of PGs on our system were also visible by TEM, highlighting the positive effect of CXL on the KC ECM. Taken together, CXL, appears to be very effective modulating PGs and therefore strengthening the corneal ECM.

Clearly, further work is warranted to determine and better understand the ultrastructural alterations caused by CXL when applied on KC corneas. Continuation of these studies will pave the way for a personalized CXL treatment that will ultimately benefit the KC patients.

## Conclusion

Our 3D cell-based CXL model is unique in that it gives us the opportunity to determine and quantify the effect of CXL at the cellular and molecular level. The most critical outcomes from the current work are: 1) CXL is effective in strengthening and organizing the collagenous ECM, as shown by our TEM data, 2) Not all PGs respond the same to CXL, highlighting the need for delineating their role in KC, 3) CXL is selectively modulating MMPs in order to arrest ECM degradation, as indicated by the MMP/TIMP data.

Conclusively, this study, paves the way for a deeper understanding on how CXL works and may lead us to otherwise unknown molecular/therapeutic targets.
